# Genetic diversity and population differentiation of small giant clam *Tridacna maxima* in Comoros islands assessed by microsatellite markers

**DOI:** 10.1186/s40064-016-3513-6

**Published:** 2016-10-22

**Authors:** Nadjim Ahmed Mohamed, Qian Yu, Mohamed Ibrahim Chanfi, Yangping Li, Shi Wang, Xiaoting Huang, Zhenmin Bao

**Affiliations:** 1 Key Laboratory of Marine Genetics and Breeding, College of Marine Life Sciences, Ocean University of China, Qingdao, 266003 China; 2 Faculty of Sciences and Technology, University of Comoros, BP 2585, Moroni Corniche, Comoros

**Keywords:** *Tridacna maxima*, Comoros islands, Genetic diversity, Population differentiation, Gene flow, Marine protected areas

## Abstract

Small giant clam, *Tridacna maxima*, widely distributed from French Polynesia to East Africa, has faced population declines due to over-exploitation. Comoros islands are an important biogeographic region due to potential richness of marine species, but no relevant information is available. In order to facilitate devising effective conservation management plan for *T. maxima*, nine microsatellite markers were used to survey genetic diversity and population differentiation of 72 specimens collected from three Comoros islands, Grande Comore, Moheli and Anjouan. A total of 51 alleles were detected ranged from 2 to 8 per locus. Observed and expected heterozygosity varied from 0.260 to 0.790 and from 0.542 to 0.830, respectively. All populations have high genetic diversity, especially the population in Moheli, a protected area, has higher genetic diversity than the others. Significant heterozygote deficiencies were recorded, and null alleles were probably the main factor leading to these deficits. *F*_ST_ value indicated medium genetic differentiation among the populations. Although significant, AMOVA revealed 48.9 % of genetic variation within individuals and only a small variation of 8.9 % was found between populations. Gene flow was high (*Nm* = 12.40) between Grande Comore and Moheli, while lower (*Nm* = 1.80) between Grande Comore and Anjouan, explaining geographic barriers to genetic exchanges might exist in these two islands. Global gene flow analysis (*Nm* = 5.50) showed that larval dispersal is enough to move between the islands. The high genetic diversity and medium population differentiation revealed in the present study offer useful information on genetic conservation of small giant clams.

## Background

The giant clam subfamily Tridacninae (Schneider and Foighil 1999) is the most widespread of the bivalves and is distributed throughout the Red sea and Indo-Pacific Ocean, from French Polynesia to East Africa (bin Othman et al. 2010). There are currently eight species from the genus *Tridacna* in the world: *Tridacna. gigas* (Linnaeus, 1758), *T. maxima* (Röding, 1798), *T. crocea* (Lamarck, 1819), *T. squamosa* (Lamarck, 1819), *T. derasa* (Röding, 1798), *T. tevoroa* (Lucas, Ledua and Braley, 1991), *T. rosewateri* (Sirenko and Scarlato, 1991), *T. costata* (Roa-Quiaoit, Kochzius, Jantzen, Zibdah and Richter, 2008) (bin Othman et al. 2010). Recently, *T. noae* was separated from *T. maxima* by their genetic and morphological description (Su et al. 2014). Among these bivalves, *T. maxima* has commonly the largest distribution range (Lucas 1988). All those giant clams are settled on the coral reef in shallow water and live in symbiotic photosynthetic with xanthophyllae algae (genus Symbiodinium) that grow in the mantle tissues (Soo and Todd 2014).

Like other marine bivalves, small giant clam species (*T. maxima*) are sedentary as adults, reproduce by broadcast spawning with high fecundity (>10^6^ eggs per female), and have pelagic larval dispersal about 9 days (Lucas 1988). Based on these aspects, population genetics studies can provide more information about the ecological interactions, larval dispersal, distribution patterns, as well as evolution of the species. To date, most of studies have been conducted on *T. maxima* about spawning (Lucas 1994; Soo and Todd 2014), larval and post-larval development (Jameson 1976), and growth (Hart et al. 1998; Smith 2011; Toonen et al. 2011). Whereas only a few studies have been done on genetic diversity and genetic structure of *T. maxima*. Indeed, genetic variations studies using allozyme analysis (Campbell et al. 1975; Laurent et al. 2002) and, recently, mitochondrial markers (Nuryanto and Kochzius 2009), have provided information on highly genetic variability, larval dispersal and also the connectivity of different sites of Indo-Pacific Ocean that can be explained by marine currents or geographic isolation (Benzie and Williams 1992a, b).

Small giant clam is listed in Appendix II of CITES (United Nations Convention on International Trade in Endangered Species of Wild Fauna and Flora) and classified as lower risk conservation dependent on the IUCN (International Union for Conservation of Nature) Red List of Threatened Species. This status indicates that the population densities have declined in a large geographical region by their overexploitation and the degradation of their natural habitat (Lucas 1994; bin Othman et al. 2010; Hui et al. 2011). It seems to be still abundant according to the population densities data in some part of countries (Australia, up to 3.83 × 10^1^ individuals per m^2^ and French Polynesia, 5.84 per m^2^, for instance, see bin Othman et al. 2010). Therefore, it is crucial to intensify the conservation efforts of marine biodiversity as well as to preserve the natural marine species for sustainable development.

**Fig. 1:**
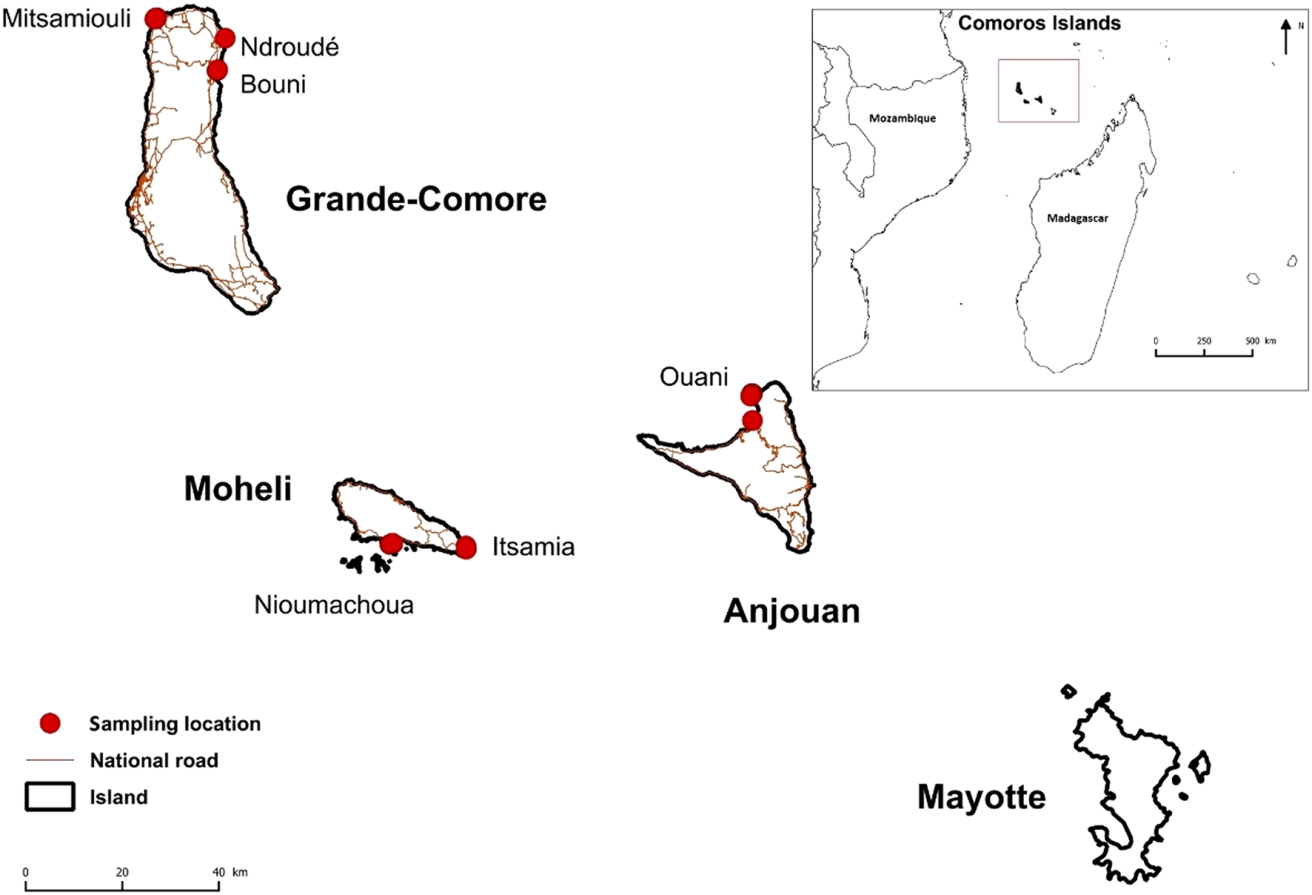
Map showing the sampling collections of *T. maxima* in Comoros islands

Comoros islands are separated from each other by a small distance, which indicate that the area is relatively narrow geographically (Fig. 1). Despite that, the area benefit a considerable interest in conservation due to the presence of abundant marine species, such as *T. maxima* but no relevant information is available to now. Recently, a research was conducted to identify and determine the marine mollusks species in Comoros islands using the photo-identification method and documentation of previous studies (Ramadhoini and Nirina, unpublished). Likewise an ecologic description have been studied on Tridacnidea family from Mayotte island (Deuss et al. 2013). Some microsatellite primers were developed from *T. maxima* by Grulois et al. (2014) and showed very high genetic diversity. In this study, we selected nine microsatellite markers (Grulois et al. 2014) to estimate the level of genetic diversity of *T. maxima* distributed in three islands of Comoros including Grande-Comore (Gc population), Anjouan (An population) and Moheli (Mo population). At the same time, we investigated the population differentiation in order to implement the conservation strategies of the *T. maxima*.

## Methods

### Sample collection and DNA extraction

Small giant clams (*n* = 72) were collected randomly between intertidal and subtidal zones at depth range of 0–20 m either by snorkeling or hand picking in three different localitions through the coral reefs of Grande-Comore (Gc), Anjouan (An) and Moheli (Mo) in June 2015 (Fig. 1; Table 1). The geographic distance between the study areas is approximatively 100, 140 and 70 km, between Gc-Mo, Gc-An, and Mo-An, respectively. For all specimens found, GPS positions were recorded and shells were measured (maximum length) using Vernier calipers.

**Table 1:** Sample details of *T. maxima*. For each sampling location, geographical coordinates, number (n) of individuals, shell length (L) and collection time are shown

Sample locality (abbreviation used)	Geographical coordinates	n	L (cm)	Collection time
Grande-Comore (Gc)	From 11°23′S and 43°17′E to 11°29′S and 43°24′E	24	16.85 ± 4.34	June 2015
Moheli (Mo)	From 12°22′S and 43°44′E to 12°22′S and 43°52′E	20	17.08 ± 3.68	June 2015
Anjouan (An)	12°05′S and 44°25′E	28	18.80 ± 5.50	June 2015

Adductor muscles were taken, rinsed and preserved in 95 % ethanol until DNA preparation. Genomic DNA was extracted following the protocol described by Zhan et al. (2009). The DNA was checked on 1 % agarose gel and the concentration was determined for each sample using NanoView spectrophotometer, afterwards stored at −20 °C prior to genetic analysis performed.

### SSR amplification and genotyping

Individual genotypes were assessed using nine microsatellite markers (Grulois et al. 2014) (Table 2). PCR amplifications were performed in a final volume of 10 μl containing 20–50 ng of genomic DNA, 10 μM of each primer, 0.2 mM dNTPs (Takara Bio Inc.), 10× PCR buffer (Takara Bio Inc.), and 0.5 U Taq DNA polymerase (Takara Bio Inc.). Reactions were carried out on a thermal cycler (Bio-Rad Laboratories, Inc.) using the following steps: an initial denaturing step at 95 °C for 5 min, followed by 35 cycles of 95 °C for 30 s, 54 °C for 45 s and 72 °C for 45 s with a final extension at 72 °C for 5 min. PCR products were electrophoresed on 10 % polyacrylamide gel using 1× TBE buffer for 1 h, stained with ethidium bromide and visualize under ultraviolet light.

**Table 2:** Respective sequences of nine microsatellite loci of *T. maxima* developed by Grulois et al. (2014) using in our study

Locus	Primer sequence 5′-3′	Size
Tm06526	F: TCCCATTGAAAAGTCTACGCAC R: GCTGCAGAAATTTGTTCGACATC	263–295
Tm11666	F: ATCGCACTTCCGCTTTGATG R: ATTTATCGTGAACCCTATATCGC	217–253
Tm14538	F: AGCCTAGAGAGAAATACAGAAAGG R: GTCTCACCGAACTAGATCCCC	88–120
Tm20025	F: GCGCGAGAAATCTAAGGCAC R: ACATCTGTAGAAAGTCTTGTTATCATC	240–282
Tm23637	F: GTCCTTGGGCAGGAGATTTTG R: ACTCTGAGGGTGTTGATTGAC	199–243
Tm23670	F: GGTCGGTAGAGAAGGTGTCC R: CCGCCTTCAAATCCATCCAC	143–217
Tm24162	F: TGGACAGATTCAGTGTCGGC R: GACCGGTTTGAATGGAGCTG	193–260
Tm24224	F: TGTATGCCGTCCACAAAAGC R: TTCGAAGAAAGTCCACACCG	258–292
Tm25349	F: TCCGTTTCCTATTGATGTTGTCC R: CATCTCTGGCGGCAGTTTG	105–133

### Data analysis

For each marker, allele number (*Na*), allele frequency, observed heterozygosity (*H*_*O*_), expected heterozygosity (*H*_*E*_), Nei’s unbiased genetic distance and genetic similarity between populations were calculated using POPGENE 1.32 (Yeh et al. 1999). Allele richness (*A*_*R*_) was carried out using FSTAT 2.9.3 (Goudet 2001). Hardy–Weinberg equilibrium (HWE) and linkage disequilibrium were conducted using or GENEPOP 4.2 program (Rousset 2008). Sequential Bonferroni correction was conducted to adjust the significant level (Holm 1979; Rice 1989). The presence of null allele was detected using MICOR-CHECKER 2.2.3 (Van Oosterhout et al. 2004). F-statistics (*F*_*IS*_, *F*_*ST*_ and *F*_*IT*_) and gene flow (*Nm*) were calculated using GENETIX 4.05. Hierarchical Analysis of Molecular Variance (AMOVA) was conducted with ARLEQUIN 3.5 (Excoffier and Lischer 2010) to investigate regional population differentiation. Cluster analysis was performed to construct dendrogram using the unweighted pair group method average (UPGMA) by MEGA 6.06.

## Results

Among 72 individuals, a total of 51 alleles were detected. The alleles number per locus ranged from 2 to 8 (mean = 5.6). Overall, Mo specimens showed the highest *H*_*O*_ and *H*_*E*_, 0.460 and 0.715, respectively. While Gc had the lowest value of *H*_*O*_ and *H*_*E*_, 0.320 and 0.695, respectively (Table 4). Specimens from Mo revealed the highest mean value of Allelic richness (*A*_*R*_ = 5.262).

Significant deviations from HWE (P < 0.05) were detected in 21 cases of the 27 locus-population combination after Sequential Bonferroni correction (Table 2). Null alleles decreased the number of significant deviations from HWE from 21 to 12 locus-population. Linkage disequilibrium was significant in only 4 out of 36 pairwise comparisons at the P < 0.05 level (Tm23637 vs Tm23670; Tm20025 vs Tm25349; Tm23637 vs Tm25349 and Tm24224 vs Tm25349), indicating virtually no linkage among loci.

F-statistics over all loci among all populations fixed the average values for *F*_*IS*_ *, F*_*ST*_ and *F*_*IT*_ at 0.460, 0.090 and 0.510, respectively. Pairwise comparison revealed that *F*_*ST*_ = 0.090 (0.05 < *F*_*ST*_ < 0.15) showed a moderate genetic differentiation among the three populations (Wright 1978) with significant level at P < 0.05 value. AMOVA analysis revealed that 48.9 % of the genetic variation originated within individuals whereas among the populations, the variation showed only 8.9 % (Table 3). The number of migrants per generation (*Nm*) placed the mean value at 5.50.

**Table 3:** Pairwise *F*_st_ values (below diagonal) and number of migrants per generation (above diagonal) among three populations of *T. maxima*

	Gc	An	Mo
Gc		1.80	12.40
An	0.120*		2.35
Mo	0.020*	0.095*	

* Significant at P < 0.05

The distance matrix showed that populations from Gc and Mo had the smallest genetic distance (0.120) and the highest genetic similarity (0.885) values, whereas Gc and An populations indicated the highest genetic distance (0.480) and the smallest genetic similarity (0.620) (Table 6). Furthermore, the unweighted pair group method average dendrogram revealed that Gc and Mo populations clustered together and An population formed one group.

## Discussion

### Genetic diversity and deviation from HWE

Previous studies have indicated a high level of genetic diversity on *T. maxima* in Indo-Pacific Ocean (Ayala et al. 1973; Campbell et al. 1975; Nuryanto and Kochzius 2009) and other species on Tridacnidae family (Kochzius and Nuryanto 2008; DeBoer and Barber 2010; Hui et al. 2011). This present study shows a high level of genetic diversity for the small giant clams (*H*_*E*_ = 0.699–0.714) within populations. Grulois et al. (2014) made the first attempt to investigate the genetic diversity of *T. maxima* using microsatellite markers, and observed a high value of expected heterozygosity (*H*_*E*_ = 0.591–0.935) in New Caledonia. Comparing these two populations of *T. maxima*, one from Comoros islands in West Indian Ocean showed lower genetic diversity than the population from New Caledonia in Pacific Ocean. Vicariance process due to Pleistocene sea level fluctuation might be the main factor to affect the genetic diversity among populations of indo-Pacific Ocean (Williams and Benzie 1998; Carpenter et al. 2011). Oceanographic conditions and limited larval dispersal distance could be also an important factors to explain the genetic variability of populations (Froukh and Kochzius 2007).

The phenomenon of heterozygote deficits relative to HWE in microsatellite survey is most common in marine bivalves (Lemer et al. 2011). Significant deviations have been reported in *T. maxima* populations (Grulois et al. 2014), also in others species of Tridacnidae family (DeBoer and Barber 2010; Hui et al. 2011; Tiavouane et al. 2014). In our study, six of the nine loci were deviated from HWE, and heterozygote deficiency was recorded for almost all loci and in all populations. Therefore, our data (positive *F*_*IS*_ values in Table 4) suggested that inbreeding might occur. Additionally, deficits of heterozygotes in HWE tests could be caused by the presence of null alleles. Among the nine loci used in this present study, four including Tm11666, Tm23637, Tm23670 and Tm24162 showed a presence of null alleles by Micro-checker analysis. Null alleles are frequently detected in many studies of marine bivalves assessed by microsatellite analysis (Gruenthal and Burton 2008) and are randomly laid to different nucleotides in primers, which are unlikely to be eliminated from all individuals (Hedgecock et al. 2004). In addition, populations of Pacific oysters (*Crassostrea gigas*) showed heterozygote deficiencies due to null alleles at microsatellites loci (Hedgecock et al. 2004), which is similar with the results of *T. maxima* (Grulois et al. 2014). Therefore, it seems likely that null alleles may be the major cause of heterozygotes deficiencies.

**Table 4:** Allele number (*N*_*A*_), observed heterozygoty (*H*_*O*_), expected heterozygoty (*H*_*E*_), allelic richness (*A*_*R*_), population inbreeding coefficient (*F*_*IS*_) and Hardy–Weinberg equilibrium (HWE)

Site (code)	GeneBank accession/locus									Mean
	KM267264	KM267265	KM267266	KM267268	KM267269	KM267270	KM267271	KM267272	KM267273	
	Tm06526	Tm11666	Tm14538	Tm20025	Tm23637	Tm23670	Tm24162	Tm24224	Tm25349	
Grande Comore (Gc)										
*N*_*A*_	6	6	5	5	5	4	5	5	2	4.777
*H*_*O*_	0.444	0.350	0.500	0.761	0.090	0.318	0.368	0.050	0.000	0.320
*H*_*E*_	0.836	0.792	0.794	0.794	0.778	0.760	0.763	0.783	0.102	0.695
*A*_*R*_	5.997	5.692	4.998	4.998	4.904	4.000	4.992	4.942	1.960	4.720
*F*_*IS*_	0.568	0.555	0.580	0.628	0.510	0.552	0.560	0.502	0.549	0.555
HWE (*P value*)	*0.000*	*0.007*	*0.008*	0.525	*0.000*	*0.000*	*0.000*	*0.000*	*0.0280*	–
Anjouan (An)										
*N*_*A*_	5	6	8	4	6	4	5	4	3	5
*H*_*O*_	0.708	0.480	0.478	0.777	0.153	0.407	0.440	0.000	0.000	0.382
*H*_*E*_	0.778	0.766	0.848	0.754	0.725	0.748	0.721	0.728	0.222	0.699
*A*_*R*_	4.953	5.444	7.419	4.000	5.228	4.000	4.883	3.998	2.824	4.750
*F*_*IS*_	0.509	0.468	0.460	0.525	0.414	0.459	0.465	0.385	0.438	0.460
HWE (*P value*)	0.143	*0.000*	*0.002*	0.692	*0.000*	*0.000*	*0.019*	*0.000*	*0.000*	–
Moheli (Mo)										
*N*_*A*_	5	7	7	5	7	4	5	5	3	5.333
*H*_*O*_	0.800	0.250	0.588	0.833	0.611	0.470	0.466	0.117	0.000	0.460
*H*_*E*_	0.802	0.681	0.714	0.804	0.850	0.768	0.726	0.736	0.349	0.715
*A*_*R*_	5.000	6.810	6.850	5.000	6.833	4.000	5.000	4.872	3.000	5.262
*F*_*IS*_	0.415	0.331	0.387	0.420	0.376	0.360	0.364	0.302	0.327	0.365
HWE (*P value*)	0.4441	*0.000*	0.270	0.479	*0.000*	*0.003*	*0.000*	*0.000*	*0.000*	–

Value in italic indicates significant deviations from HWE (P < 0.05) after sequential Bonferroni corrections

### Genetic differentiation among populations

**Fig. 2:**
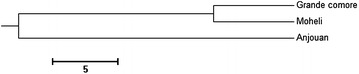
Unweighted pair group method average dendrogram (UPGMA) based on Nei’s Genetic distance among population

*F*_*ST*_, *Nm* and genetic distance are commonly used to measure the genetic differentiation. Indeed, our data showed that genetic differentiation was moderate among all populations from global pairwise *F*_*ST*_ value. AMOVA also indicated that most variation is attributed to genetic difference within individuals (48.9 %), while variation among populations is low, accounting for only 8.9 % of the total variations (Table 5). Hence, it is indicating the presence of genetic heterogeneity among these three populations. Therefore, the assumption of panmixia was rejected among all populations. In addition, our data have revealed an average gene flow value (5.51) greater than 1, confirming genetic drift is not the factor to explain the genetic exchanges between these populations (Slatkin 1985). Therefore, due to the sedentary of *T. maxima* such as others marine bivalves, larval dispersal can be the main factor influencing gene flow and population differentiation. Although it was demonstrated that *T. maxima* have pelagic larvae dispersal about 9 days (Lucas 1988) to travel long distance about 500 km, which means that population differentiation should become detectable among the three islands (approximately 80 km between them). However, gene flow along the dispersal route between Gc and An islands is lower than that between Gc and Mo islands, and also Mo and An islands (Table 4). It indicates limited larval dispersal and geographic barriers like marine currents restricted gene exchanges among these islands. Additionally, the topology of the UPGMA tree (Fig. 2) and the genetic distance (Table 6) also suggested that gene flow between Gc and An populations was limited and barriers to genetic exchanges might exist among these two populations. Moreover, another possible reason to explain the high gene flow and the clustering between the populations of Gc and Mo could be caused by the angling boats traffic massive moving between the two lands, suggesting the high larval dispersal.

**Table 5:** AMOVA analysis for three populations of *T. maxima*

Source of variation	d.f.	Sum of squares	Variance components	Percentage of variation
Among populations	2	34.151	0.315	8.9*
Among individuals within populations	67	278.063	1.491	42.2*
Within individuals	70	107.000	1.727	48.9*
Total	139	419.214	3.534	

* Significant at P < 0.05

**Table 6:** Nei’s unbiased genetic similarity (above diagonal) and genetic distance (below diagonal)

	Gc	An	Mo
Gc		0.620	0.885
An	0.480		0.690
Mo	0.120	0.350	

### Implication for conservation

Tridacnid species are listed in Appendix II of CITES and are classified as vulnerable on the IUCN Red List of Threatened Species due to their extreme exploitation for the food and marine ornamental trade. According to the CITES data, international trade of Giant clams non-captive bred increased from about 40,000 to 100,000 individuals between 1993 and 2001 (Wabnitz et al. 2003). In Comoros islands, despite the existence of the legislations for marine resources, *T. maxima* were especially exploited for food and their big shells were used for different purposes such as ornamental objects. Furthermore, over-exploitation, pollution, reef degradation by trampling or destructive fishing practices, and coral bleaching event due to rising sea temperature by El-nino event in 1997/1998 are likely to lead negative effects (ASCLME 2012). Therefore, one protected area (Moheli Marine Park) covering a total area of 403.6 km^2^ was created in Comoros to ensure the sustainable use of living marine resources (Beudard 2003).

In our study, the genetic diversity in the three islands showed that population from Mo (*H*_*E*_ = 0.714; *A*_*R*_ = 5.26) is higher than Gc and An (*H*_*E*_ = 0.694; *A*_*R*_ = 4.720, *H*_*E*_ = 0.699; *A*_*R*_ = 4.75), respectively. The protection of the area could be the major factor to explain high genetic diversity in Mo population. As there is a Marine national park in Moheli (Beudard 2003), species in the island benefit from its protection. Compared to the others islands where there are not restricted of any specific protection, Moheli is genetically more diverse, which can play an important role for allele distribution in the others islands. Therefore, Moheli Marine Park is most probably insufficient for the protection of *T. maxima*. While they have a larval dispersal time about 9 days, specimens of *T. maxima* are able to travel a distance about 500 km. Even though the populations between Gc and An showed low larval dispersal, it is possible to detect a connectivity for populations among the three islands from Comoros because of their small scale area. Therefore, further studies based on oceanographic barriers and ecological barriers in addition to genetic data are more important to understand the marine organism movements and connectivity between the islands. The genetic diversity and population differentiation of *T. maxima* can offer useful information to establish an effective plan for conservation management.
