# Erratum to: Genetic diversity and population differentiation of small giant clam *Tridacna maxima* in Comoros islands assessed by microsatellite markers

**DOI:** 10.1186/s40064-016-3684-1

**Published:** 2016-11-28

**Authors:** Nadjim Ahmed Mohamed, Qian Yu, Mohamed Ibrahim Chanfi, Yangping Li, Shi Wang, Xiaoting Huang, Zhenmin Bao

**Affiliations:** 1 Key Laboratory of Marine Genetics and Breeding, College of Marine Life Sciences, Ocean University of China, Qingdao, 266003 China; 2 Faculty of Sciences and Technology, University of Comoros, BP 2585, Moroni Corniche, Comoros

## Erratum to: SpringerPlus (2016) 5:1852 DOI 10.1186/s40064-016-3513-6

Upon publication, it was noticed that in the original version of the article (Ahmed Mohamed et al. 2016), the referencing of several tables was mislabelled.

1. In the Results section, for sentence: “Significant deviations from HWE (P < 0.05) were detected in 21 cases of the 27 locus-population combination after sequential Bonferroni correction”, “Table 4” should be referenced instead of “Table 2”.
2. Also in the Results section, for sentence: “AMOVA analysis revealed that 48.9% of the genetic variation originated within individuals whereas among the populations, the variation showed only 8.9%”, “Table 5” should be referenced instead of “Table 3”.
3. In the Discussion section, for sentence: “However, gene flow along the dispersal route between Gc and An islands is lower than that between Gc and Mo islands, and also Mo and An islands”, “Table 3” should be referenced instead of “Table 4”.
